# Does personalised nutrition advice based on apolipoprotein E and methylenetetrahydrofolate reductase genotype affect dietary behaviour?

**DOI:** 10.1177/02601060211032882

**Published:** 2021-11-24

**Authors:** Alexandra King, Shaghayegh Saifi, Jenna Smith, Leta Pilic, Catherine A-M Graham, Viviane Da Silva Anastacio, Mark Glaister, Yiannis Mavrommatis

**Affiliations:** 1 Faculty of Sport, Allied Health and Performance Science, 62693 St Mary’s University Twickenham, UK; 2 Faculty of Health and Life Sciences, Department of Sport, Health and Social Work, Oxford Brookes Centre for Nutrition and Health, 98464 Oxford Brookes University, UK

**Keywords:** Apolipoprotein E, methylenetetrahydrofolate reductase, dietary folate, dietary fat, personalised nutrition, behaviour change

## Abstract

**Background:** Dietary intake is linked to numerous modifiable risk factors of cardiovascular disease. Current dietary recommendations in the UK to reduce the risk of cardiovascular disease are not being met. A genotype-based personalised approach to dietary recommendations may motivate individuals to make positive changes in their dietary behaviour. **Aim:** To determine the effect of a personalised nutrition intervention, based on apolipoprotein E (*ApoE*, rs7412; rs429358) and methylenetetrahydrofolate reductase (*MTHFR*, rs1801133) genotype, on reported dietary intake of saturated fat and folate in participants informed of a risk genotype compared to those informed of non-risk genotype. **Methods:** Baseline data (*n* = 99) were collected to determine genotype (non-risk vs risk), dietary intake and cardiovascular risk (Q-Risk®2 cardiovascular risk calculator). Participants were provided with personalised nutrition advice via email based on their *ApoE* and *MTHFR* genotype and reported intake of folate and saturated fat. After 10 days, dietary intake data were reported for a second time. **Results:** Personalised nutrition advice led to favourable dietary changes, irrespective of genotype, in participants who were not meeting dietary recommendations at baseline for saturated fat (*p* < 0.001) and folate (*p* = 0.002). Only participants who were informed of a risk *ApoE* genotype met saturated fat recommendations following personalised nutrition advice. **Conclusion:** Incorporation of genotype-based personalised nutrition advice in a diet behaviour intervention may elicit favourable changes in dietary behaviour in participants informed of a risk genotype. Participants informed of a non-risk genotype also respond to personalised nutrition advice favourably but to a lesser extent.

## Background

Cardiovascular disease (CVD) is the most common cause of death worldwide, causing an estimated 17.9 million deaths globally (Wang et al., 2016). In the United Kingdom (UK) CVD is the second most common cause of death after cancer, causing approximately 168,000 deaths in 2017 (British Heart Foundation, 2019). CVD is a preventable cause of premature death and dietary intake is linked to numerous modifiable risk factors of CVD (NICE, 2010). A recent survey of the UK population suggests that current dietary advice to reduce the risk of CVD is not being met (British Heart Foundation, 2017; Roberts et al., 2018). In contrast to current public health dietary recommendations which use a ‘one size fits all’ approach, it has been suggested that a gene-based personalised approach to dietary recommendations may motivate individuals to make positive changes in their dietary behaviour (Celis-Morales et al., 2015a).

There is evidence to suggest that single nucleotide polymorphisms (SNPs) in the apolipoprotein E (*ApoE*) gene rs7412 (E2) rs429358 (E4) and in the methylenetetrahydrofolate reductase (*MTHFR*) gene rs1801133 (C/T) are associated with CVD risk; this evidence can be used to provide more effective dietary advice at the individual or genetic subgroup level (Grimaldi et al., 2017). A positive dose response has been reported between *ApoE* genotype and low-density lipoprotein (LDL) cholesterol, with the lowest concentrations in E2/E2 carriers and the highest concentrations in E4/E4 (Khan et al., 2013). Consequently, reduced saturated fat intake has been suggested as a means of reducing CVD risk in individuals with an ApoE4 genotype (Minihane et al., 2007). A common missense SNP of the *MTHFR* gene affects the thermostability of the corresponding enzyme (Frosst et al., 1995). Hyperhomocysteinaemia has been identified as a risk factor for CVD. Reduced *MTHFR* activity results in increased plasma homocysteine levels and reduced plasma folate levels in TT homozygotes (Liew and Gupta, 2015).

To date, studies investigating the effect of genotype-based personalised nutrition advice on dietary behaviour have reported mixed findings. Compared to a control group, participants with a risk-associated genotype significantly improved the fat quality of their diet (Hietaranta-Luoma et al., 2014), reduced sodium (Nielsen and El-Sohemy, 2014), fat (Horne et al., 2020) and saturated fat intake (Fallaize et al., 2016), improved their adherence to a Mediterranean diet (Livingstone et al., 2016), were more likely to maintain weight loss (Arkadianos et al., 2007; Vranceanu et al., 2020) and were more likely to make health behaviour changes to reduce Alzheimer's disease (AD) risk (Chao et al., 2008). In contrast, findings of no effect have been reported in response to advice to increase folate intake (O’Donovan et al., 2016), diabetes risk (Grant et al., 2013) and a weight loss programme (Frankwich et al., 2015). Hollands et al. (2016) analysed seven randomised controlled trials and reported no significant evidence of a benefit of DNA-based risk communication on dietary behaviour change, with an OR of 0.12 (CI: 0.00–0.24).

Comparisons have also been made between participants informed of a risk-associated genotype and those informed of a non-risk-associated genotype. Participants informed of an *ApoE* risk associated genotype have been reported to make greater changes to saturated fat intake (Fallaize et al., 2016) and made and maintained moderate changes to dietary behaviour which resulted in slight improvements in clinical CVD markers 5.5–6.5 years after disclosure, (Hietaranta-Luoma et al., 2018) compared to participants informed of a non-risk genotype. However, there was no significant difference in folate intake between participants informed of an *MTHFR* risk associated genotype and those informed of a non-risk associated genotype, following a recommendation to increase their folate intake (O’Donovan et al., 2016). The aim of disclosure of genetic risk is to motivate behaviour change in these individuals, however it is also of importance to consider the effect of disclosure of a non-risk genotype which has the potential to reduce compliance to health behaviours (Lovegrove and Gitau, 2008).

Unanswered questions remain regarding the efficacy of genotype-based personalised nutrition as an intervention for positive dietary behaviour change. Furthermore, the effect of disclosure of a non-risk as well as a risk associated genotype on dietary behaviour warrants further investigation. The present study therefore used behaviour change techniques (BCTs) in the context of two SNPs with strong evidence of an interaction with dietary behaviours that affect CVD risk to motivate positive changes in related dietary behaviours. The aim of the present study was to determine the effect of personalised nutrition advice based on *ApoE* and *MTHFR* genotype on dietary intake of saturated fat and folate in participants informed of a risk genotype compared to those informed of non-risk genotype.

## Methods

### Study population

Men and women (aged ≥ 18 years) without a current diagnosis of coronary heart disease (including angina or heart attack) or stroke/transient ischaemic attack were recruited to take part in the study. Participants were recruited through advertisements and internet postings. Baseline data were collected from 114 participants, 99 participants completed the study.

This study was conducted according to the guidelines laid down in the Declaration of Helsinki and all procedures involving human subjects/patients were approved by the Institutional Ethical Committee. Written informed consent was obtained from all participants. All data were collected and stored according to the Data Protection Act 1998 and the Human Tissue Authority. This study is registered at researchregistry.com.

### Study design

**Figure 1.**
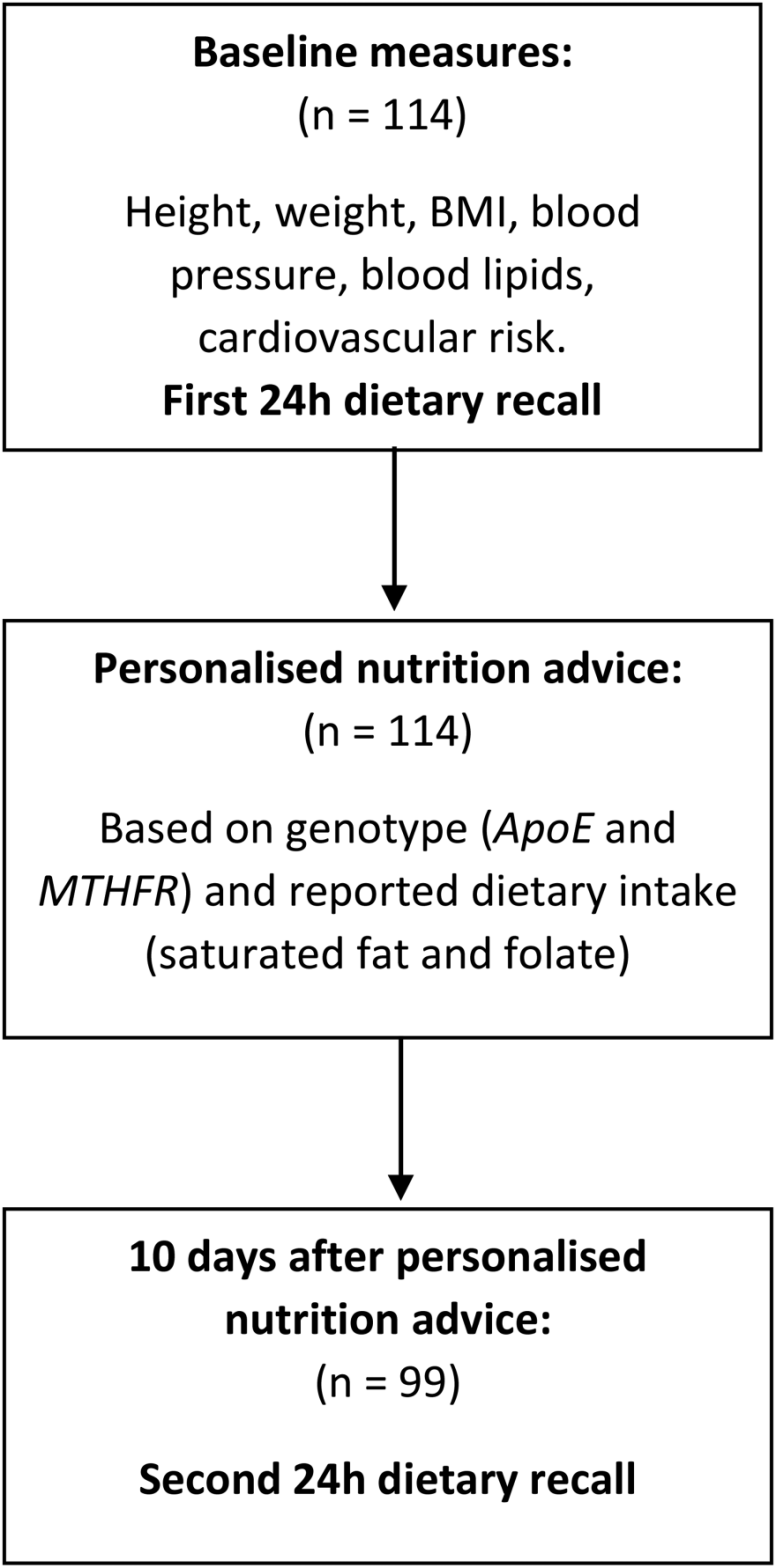
Study design flowchart.

BMI: body mass index; ApoE: apolipoprotein E; MTHFR: methylenetetrahydrofolate reductase.

Baseline measures were collected in person and included participants’ height, weight, blood pressure, blood lipids, dietary intake and 10-year cardiovascular risk. A saliva sample was obtained for genotyping. Following genotyping, participants were provided with gene-based personalised nutrition advice via email and 10 days after receiving this advice they were asked to complete a second 24-h dietary recall (Figure 1).

### Baseline measures

Height was measured without shoes using a free-standing height measure (Seca UK, Birmingham, UK). Weight was measured clothed without shoes or overgarments using a portable scale (MPMS-230 Marsden Weighing Group, Oxfordshire UK). Body mass index (BMI) was calculated by dividing participants’ weight (kg) by their height (m) squared. Blood pressure was measured for each participant using a digital blood pressure monitor (OMRON i-C10, OMRON Healthcare Europe B.V. Hoofddorp, Netherlands) on both arms. Total cholesterol (TC), high-density lipoprotein cholesterol (HDL) and triglycerides were measured from a capillary blood sample using a point-of-care test system (The CardioChek® Professional Analyser, Polymer Technology Systems Inc., Indianapolis, USA) in accordance with the manufacturers’ protocol. Cardiovascular risk was estimated using the QRISK®2-2017 CVD risk calculator.

### Dietary intake

Habitual dietary intake was estimated from a 24-h recall, administered as an online survey, using the multiple-pass approach (Moshfegh et al., 2008). Reported dietary intake data (including dietary supplements) were analysed using nutrition analysis software (Nutritics; Nutritics Ltd, Swords, Ireland), to determine energy, saturated fat and folate intake.

### Genotype-based personalised nutrition advice

Participants were provided with personalised nutrition advice based on their *ApoE* genotype and *MTHFR* genotype by email. For *ApoE,* a risk-associated genotype was defined as the presence of an E4 allele (E3/E4 and E4/E4) and for *MTHFR* a risk-associated genotype was defined as the presence of a T allele (CT and TT). To improve the reporting, implementation and evaluation of behaviour change interventions, Michie et al. (2011) developed a taxonomy of BCT for physical activity and healthy eating behaviours, four of which lend themselves to a genotype-based personalised nutrition intervention delivered via email. Firstly, participants were informed for both genotypes whether they had a risk-associated genotype. The framing of this information was designed to promote ‘*fear arousal*’ as a BCT, for example, for *MTHFR,* those with a risk-associated genotype were informed ‘You have a genetic variation in the *MTHFR* gene that is associated with a higher CVD risk; consequently, it is beneficial for you to keep a healthy intake of folate’. This also highlights the ‘*consequences of their dietary behaviour to them as an individual*’. Conversely, participants with a non-risk genotype were advised to follow healthy eating guidelines as recommended in the Eatwell Guide. Participants were informed of their dietary intake of folate and saturated fat and whether they were meeting current UK recommendations (folate >200 µg/day; saturated fat <11% TEI) (Department of Health, 1991). Therefore, participants were encouraged to make a behavioural resolution (‘*goal setting*’) to change their dietary behaviour in order to meet dietary recommendations. Finally, participants were provided with advice on how they could increase their folate intake and reduce their saturated fat intake, therefore ‘*how to perform the behaviour*’.

### DNA isolation and genotyping

Genotyping was performed according to a method described elsewhere (Pilic and Mavrommatis, 2018). In brief, genotyping for *ApoE* genotype rs7412 (E2) rs429358 (E4) and *MTHFR* genotype C677T rs1801133 was carried out using the TaqMan® method using qPCR (StepOnePlus Real-time, LifeSciences, Applied Biosystems, CA, USA) with two technical replicates for each sample. The primers and the probes were pre-designed by Applied Biosystems with the following codes; C_904973_10; C_3084793_20; C_1202883_20. The polymerase chain reaction amplification was performed under the conditions specified by the manufacturer. Genotypes were inferred by Thermofisher Connect™ platform. Call rates for all SNPs were above 95%. Genotype frequencies were within Hardy Weinberg Equilibrium for rs1801133 in the *MTHFR* gene (*p* = 0.904) and for rs7412 in the *ApoE* gene (*p* = 0.760) but not for rs429358 in the *ApoE* gene (*p* = 0.037). However, haplotype frequencies (ɛ2, 6%; ɛ3, 82%; ɛ4, 12%) and participant profiles were similar to previous studies (Fallaize et al., 2016; Schiele et al., 2000).

### Statistical analysis

A sample size of 110 was calculated based on a decrease in saturated fat intake by 2% of total energy intake in the ApoE risk group (expected ratio of non-risk to risk of 7:3, 1−*β* = 0.8, *α* = 0.05 and standard deviation (SD) = 3.4 g/day). The sample size calculation was conducted using the statistical power analyses software G*Power version 3.1.9.2 (Faul et al., 2007). Statistical analysis was carried out using IBM SPSS Statistics 24 for Windows (IBM Corp, New York, USA). The hypotheses were specified before the data were collected. The analytic plan was pre-specified and any data-driven analyses are clearly identified and discussed appropriately. Saturated fat intake was analysed as a percentage of total energy intake and folate as µg per 10 MJ. Measures of centrality and spread are presented as means ± SD. Normality of data was assessed using the Shapiro-Wilk test and if data were not normally distributed, where appropriate, it was transformed to enable parametric statistical analysis. A three-way mixed ANOVA was carried out to assess differences between genotypes (non-risk vs risk), meeting recommendations (met vs not met at baseline) and time (pre vs post advice) on reported dietary intake of saturated fat and folate. Interactions between all independent variables were also investigated. Post hoc pairwise comparisons were performed with Bonferroni corrections as appropriate. One sample *t*-tests were carried out to compare actual with recommended saturated fat intakes (Department of Health, 1991). All tests were two-tailed and considered statistically significant when *p* < 0.05.

## Results

### Participant characteristics

Baseline data including participant characteristics (age, height, weight, BMI) and intermediate CVD risk factors (systolic blood pressure, TC, HDL, TC: HDL and QRISK) were determined for 117 participants; two participants subsequently withdrew from the study and the single *ApoE* E2/E4 participant was removed from analysis because of their low population frequency. The study population was predominantly Caucasian (76%; *n* = 87). Baseline characteristics are presented in Table 1 for males and females, and Table 2 for genotype; there were no statistically significant differences in baseline characteristics of participants with a risk associated genotype compared to those with a non-risk genotype.

**Table 1.** Baseline characteristics of male and female participants.

	All (*n* = 114)	Male (*n* = 35)	Female (*n* = 79)
Age (years)	36 ± 11	36 ± 10	36 ± 12
Height (m)	1.69 ± 0.10	1.80 ± 0.08*	1.65 ± 0.06
Weight (kg)	71 ± 15	85 ± 12*	65 ± 12
BMI (kg/m^2^)	24.7 ± 4.0	26.3 ± 3.5*	24.0 ± 4.0
SBP (mmHg)	118 ± 16	128 ± 14*	113 ± 15
TC (mmol/L)	4.52 ± 0.95	4.27 ± 1.08	4.63 ± 0.87
HDL (mmol/L)	1.71 ± 0.54	1.36 ± 0.46*	1.87 ± 0.51
TC: HDL	2.90 ± 1.19	3.49 ± 1.61*	2.64 ± 0.83
Qrisk (%)	1.70 ± 3.02	2.99 ± 4.73*	1.12 ± 1.57

BMI: body mass index; SBP: systolic blood pressure; TC: total cholesterol; HDL: high-density lipoprotein cholesterol.

Values presented as means ± standard deviations. For non-normally distributed variables analysis was conducted on log-transformed values. Independent *t*-test was used to compare between males and females.

Asterisk denotes a significant difference (*p* < 0.05).

**Table 2.** Baseline characteristics of participants for genotype.

	All (*n* = 114)	*ApoE* risk (E4 + ) (*n* = 23)	*ApoE* non-risk (E4 − ) (*n* = 91)	*MTHFR* risk (CT/TT) (*n* = 53)	*MTHFR* non-risk (CC) (*n* = 61)
Gender (M/F)	35/59	6/17	29/62	14/39	21/40
Age (years)	36 ± 11	33 ± 12	37 ± 11	36 ± 11	37 ± 12
Height (m)	1.69 ± 0.10	1.70 ± 0.09	1.69 ± 0.10	1.69 ± 0.09	1.70 ± 0.10
Weight (kg)	71 ± 15	71 ± 16	71 ± 16	69 ± 13	73 ± 16
BMI (kg/m^2^)	24.7 ± 4.0	24.5 ± 3.4	24.7 ± 4.2	24.2 ± 3.7	25.1 ± 4.2
SBP (mmHg)	118 ± 16	116 ± 18	118 ± 16	116 ± 17	119 ± 15
TC (mmol/L)	4.52 ± 0.95	4.52 ± 0.96	4.52 ± 0.96	4.50 ± 0.98	4.54 ± 0.94
HDL (mmol/L)	1.71 ± 0.54	1.79 ± 0.58	1.69 ± 0.54	1.80 ± 0.57	1.64 ± 0.52
TCHDL	2.90 ± 1.19	2.76 ± 1.00	2.94 ± 1.24	2.69 ± 0.87	3.08 ± 1.39
Qrisk (%)	1.70 ± 3.02	0.95 ± 1.27	1.88 ± 3.31	1.33 ± 2.22	2.00 ± 3.58

BMI: body mass index; SBP: systolic blood pressure; TC: total cholesterol; HDL: high-density lipoprotein cholesterol; *ApoE:* apolipoprotein E; *MTHFR*: methylenetetrahydrofolate reductase.

Values presented as means ± standard deviations. For non-normally distributed variables analysis was conducted on log-transformed values. Independent *t*-test was used to compare between risk and non-risk groups, except for gender where χ^2^ analysis was used. There were no significant differences between groups.

### Effects of genotype-based personalised advice on dietary intake of saturated fat

Personalised genotype-based advice did not affect saturated fat intake in participants with a risk genotype who were meeting the saturated fat intake recommendation (*n* = 12) (*p* = 0.126). However, risk participants who were not meeting the saturated fat recommendation (*n* = 9) reduced their reported saturated fat intake following genotype-based personalised nutrition advice (*p* = 0.012).

Participants with a non-risk genotype who were meeting the saturated fat intake recommendation (*n* = 38) at baseline increased their saturated fat intake following personalised nutrition advice (*p* = 0.007), whereas participants with a non-risk-associated genotype who were not meeting the recommendation (*n* = 40) reduced their reported saturated fat intake (*p* = 0.001).

### Effects of personalised advice on meeting the recommendation for saturated fat

**Figure 2.**
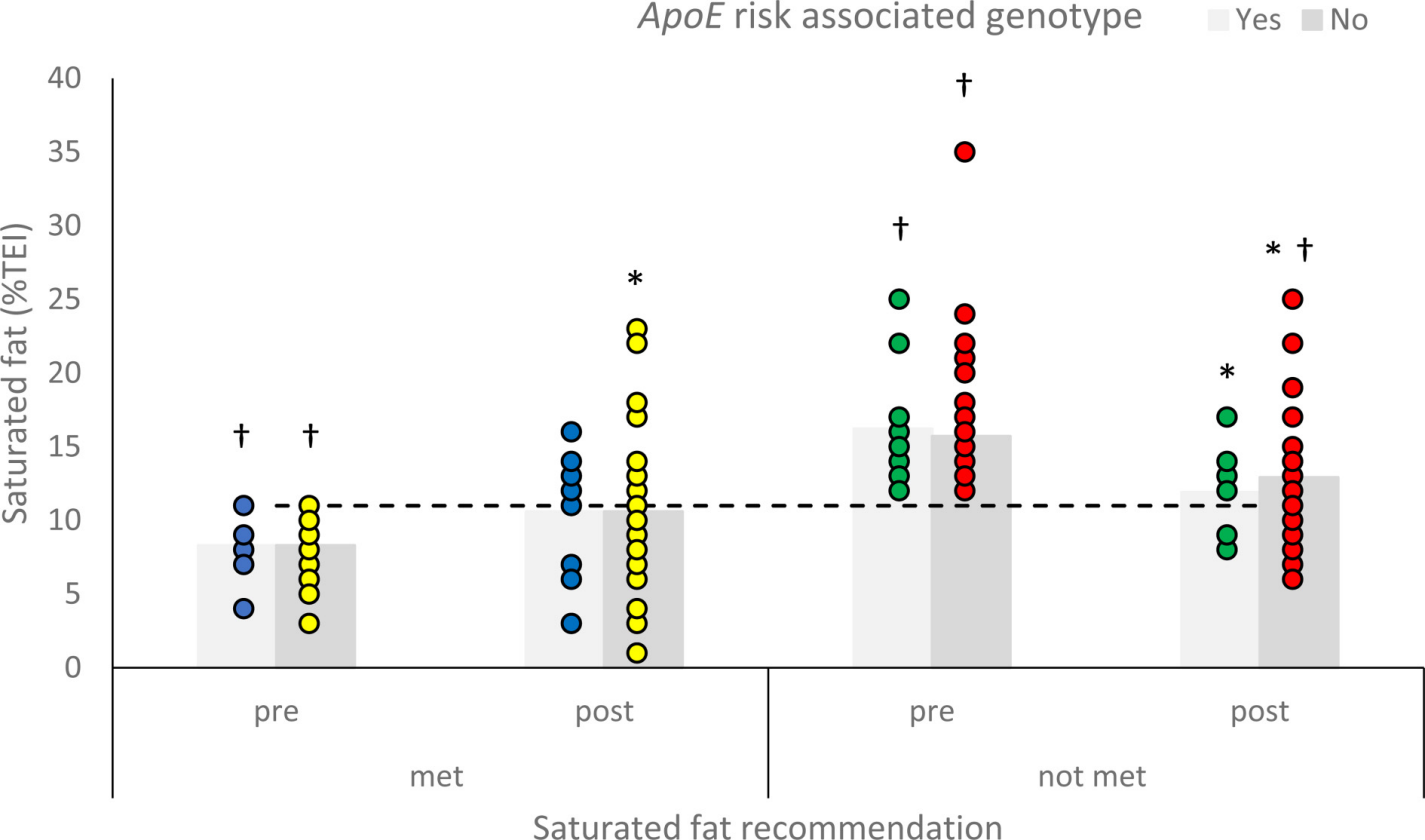
Mean reported saturated fat intake (%TEI) of participants with a risk associated or non-risk associated genotype for apolipoprotein E (ApoE), who were meeting or not meeting the saturated fat intake recommendation, before and after personalised nutrition advice. Asterisk significantly different to pre-intake (*p* < 0.05) and dagger significantly different to saturated fat recommendation (*p* < 0.05). A horizontal line indicates recommended intake (Department of Health, 1991).

In the group of participants who did not meet the saturated fat recommendation, both genotype sub-groups were above the recommended level at baseline (*p* = 0.001 for risk-associated (*n* = 11) and *p* < 0.001 for non-risk-associated (*n* = 46)). After the intervention, participants who did not meet the saturated fat recommendation at baseline and had a risk-associated genotype (*n* = 9), reduced their saturated fat intake to meet the recommendation (mean = 11.9%TEI; *p* = 0.409); however, saturated fat intakes of those without a risk-associated genotype (*n* = 40) remained significantly above the recommendation (mean = 12.9%TEI; *p* = 0.007). Both genotype groups that met the recommended intake of saturated fat before the intervention continued to meet the recommendation post-intervention (Figure 2).

### Effects of genotype-based personalised advice on dietary intake of folate

Participants with a risk genotype who were meeting the folate intake recommendation (*n* = 35) did not significantly change their folate intake following personalised nutrition advice (*p* = 0.127). In contrast, those who were not meeting the recommendation (*n* = 9) significantly increased their reported folate intake following personalised nutrition advice (*p* = 0.009).

**Figure 3.**
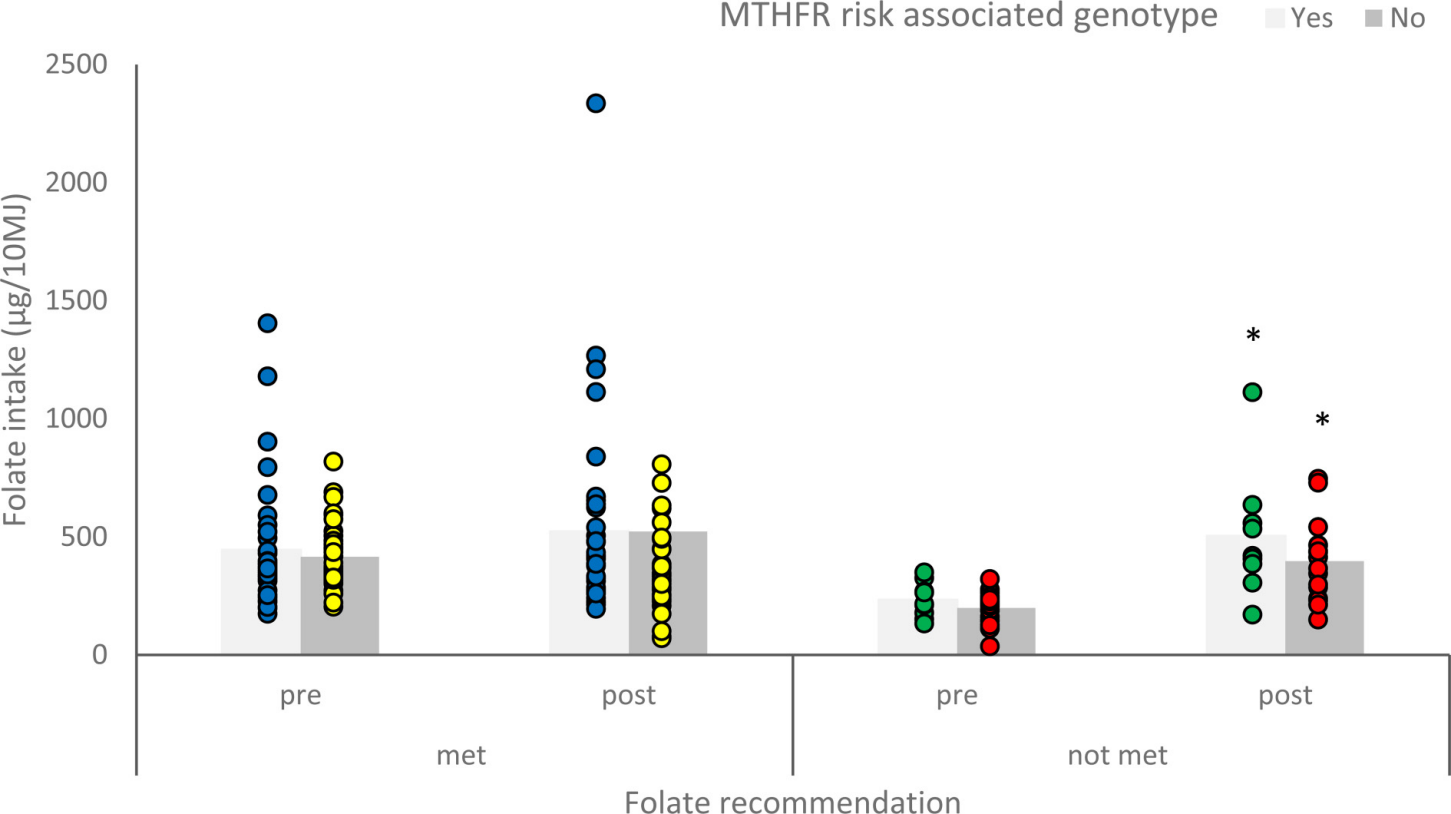
Mean reported folate intake (µg/10 MJ) of participants with a risk or non-risk associated genotype for methylenetetrahydrofolate reductase (MTHFR), who were meeting or not meeting the folate intake recommendation, before and after personalised nutrition advice. Asterisk significantly different to pre-intake (*p* < 0.05).

For participants with a non-risk genotype, those who were meeting folate intake recommendation (*n* = 39) did not significantly change their folate intake following personalised nutrition advice (*p* = 0.203), whereas those who were not meeting the recommendation (*n* = 16) significantly increased their reported folate intake following personalised nutrition advice (*p* = 0.010) (Figure 3).

## Discussion

The aim of the present study was to determine the effect of personalised nutrition advice based on *ApoE* and *MTHFR* genotype on dietary intake of saturated fat and folate in participants informed of a risk genotype compared to those informed of a non-risk genotype.

### Effects of genotype-based personalised advice on dietary intake

The main findings were that from participants that exceeded the recommended intake for saturated fat, only the group informed of genetic risk decreased their mean intake to the recommended level. The group of participants with intakes that exceeded the recommendation but were informed of non-risk genotype decreased their intake, but mean intake remained above the recommended level. Furthermore, individuals whose baseline saturated fat intakes met the recommendation, increased their saturated fat intake, although this was only significant in the non-risk group it is likely due to lower participant numbers in the risk group. Both genotype groups maintained a saturated fat intake that met the recommendation. These findings suggest that providing personalised nutrition advice based on *ApoE* genotype (incorporating BCT), promotes positive changes in dietary saturated fat intake for groups not meeting the recommendation and that the magnitude of the effect is increased in those informed of genetic risk. Participants who were not meeting the folate recommendation at baseline and were advised of a genetic risk subsequently increased their intake, as did participants who were informed that they did not have a risk-associated genotype. Similarly, participants who were meeting the folate recommendation did not change their folate intake, irrespective of their genetic risk.

*ApoE* and *MTHFR* genotype were two of five genes for which 1607 participants received genotype-based personalised nutrition advice in the Food4Me project (Celis-Morales et al., 2015b). Reported responses of genotype-based personalised nutrition advice on dietary behaviour varied depending on the gene and dietary response analysed. In agreement with the present study, intakes of saturated fat were significantly decreased in participants informed of a risk *ApoE* genotype compared to the control group, although this was also observed for participants without a risk associated genotype (Fallaize et al., 2016). However, in the present study, only the participants informed of a risk-associated genotype reduced their saturated fat intake to meet the recommended intake. Similarly, our findings for *MTHFR* genotype showed that folate intake increased in participants informed that they were not meeting the recommendation irrespective of genotype. However, O’Donovan et al. (2016), report no significant difference in folate intake after 6 months between control and risk *MTHFR* genotype group advised to increase their folate intake. Since participants in the Food4Me project received information regarding five different genotypes the effect of receiving a risk diagnosis for one genotype may have been minimised by the effect of a non-risk for another, making it more difficult to make comparisons within each genotype and corresponding health behaviour (Meisel et al., 2012). Overall dietary behaviour in the Food4Me participants was assessed by adherence to the Mediterranean diet (MedDiet score). All levels of personalisation of advice resulted in significantly greater improvements in MedDiet score compared to the control group. Furthermore, the greatest improvements were observed in participants receiving gene-based personalised nutrition advice (Livingstone et al., 2016).

This inconsistent pattern in the effect of genotype-based personalised nutrition advice on behaviour is evident from other research (Frankwich et al., 2015; Grant et al., 2013; O’Donovan et al., 2016). A meta-analysis of seven studies investigating the effect of DNA-based risk estimates on dietary behaviour change reported an odds ratio of 0.12 (confidence interval (CI): 0.00–0.24) (Hollands et al., 2016). The contradictory findings reported above may reflect the heterogeneous study designs used; the delivery of the genotype-based personalised nutrition intervention has varied between studies from remote delivery of information via email (Celis-Morales et al., 2015b) to delivery as part of a 12-week intervention programme (Grant et al., 2013). Studies have been carried out in the context of different chronic diseases and related genes, dietary behaviour has been measured in different ways with variable durations of follow up and the study participants have ranged from interested volunteers (Celis-Morales et al., 2015b) to those with a family history of a disease (Chao et al., 2008). Participants of the present study were generally in good health with baseline blood pressure, cholesterol and QRISK2 scores of the study participants suggesting that they were on average at low risk of CVD (NICE, 2014). However, by volunteering to take part in the study they demonstrated an interest in their health and genotype-based personalised nutrition.

The incorporation of behaviour change theory in genetic-based lifestyle behaviour interventions has been suggested as a way to improve efficacy (Horne et al., 2018; NICE, 2007). In the present study, the framing of genetic information to the participant was designed to promote ‘fear arousal’, to make the participant fearful of the risk of developing CVD to motivate behaviour change (Wilson, 2007). This BCT was not incorporated in the Food4Me project and was suggested as an explanation for not observing a significant difference between participants with an *ApoE* risk genotype compared to those with a non-risk genotype (Fallaize et al., 2016). The framing of the message to participants in the REVEAL study, as in the present study, was designed to promote ‘fear arousal’ and they reported, participants with a risk-associated genotype were more likely to make AD-related health behaviour changes than those without a risk associated genotype or control (Chao et al., 2008).

### Public health application

In line with our findings, previous studies have reported significant positive changes in health behaviour in participants informed of a high *ApoE* genetic risk in the context of CVD or AD (Chao et al., 2008; Fanshawe et al., 2008; Hietaranta-Luoma et al., 2014, 2018; Vernarelli et al., 2010). A significant effect of genotype-based personalised nutrition advice has also been reported for genes related to sodium intake (Nielsen and El-Sohemy, 2014) and weight loss (Arkadianos et al., 2007; Vranceanu et al., 2020). Dietary recommendations in the UK are not being met, with mean intakes of saturated fat exceeding recommendations in all age groups studied (Roberts et al., 2018). Public health interventions appear to raise population awareness but fail to translate into the modification of behaviour (Croker et al., 2012). One factor that has been suggested to explain the lack of response to public health campaigns to encourage healthy behaviours is ‘optimistic bias’; the phenomenon by which an individual underestimates their own risk of developing a disease, such as CVD, compared to others (Shepherd, 1999). Genotype-based personalised dietary advice enables the personal salience of dietary advice to be highlighted to those with a risk-associated genotype. Personal salience of health advice is more difficult to achieve with a ‘one size fits all’ approach and has been identified as a key concept in the delivery of behaviour change interventions (NICE, 2007).

Making dietary information personally salient to participants with a risk-associated genotype, could increase optimistic bias for participants with a non-risk associated genotype (Hunter et al., 2008). The findings of the present study suggest that the pattern of dietary change is similar for participants with risk and non-risk genotype. This is in accordance with findings of previous studies, non-risk participants not meeting recommendations still make positive dietary behaviour changes, although they may be smaller than those in participants without knowledge of their genotype (Fallaize et al., 2016; Nielsen and El-Sohemy, 2014), which highlights the importance of how nutrigenetic advice is disclosed to participants (Nielsen and El-Sohemy, 2014). In reality, individuals seeking advice from nutrigenetic testing companies will receive information about a panel of genes, some of which are likely to be risk conferring and others protective. Therefore, the receipt of this information alongside dietary advice is likely to be received in a balanced way.

### Strengths and limitations

A strength of the present study was the successful collection of dietary information and delivery of health advice via email. The importance of remote delivery of health interventions has been highlighted during the COVID-19 pandemic and acceptability of this mode of delivery may increase (Martin et al., 2020). Dietary intake was measured rather than participants reporting if they had changed their behaviour (Chao et al., 2008; Fanshawe et al., 2008; Vernarelli et al., 2010) or their intention to change their behaviour (Grant et al., 2013). However, the measurement of habitual dietary intake is a major challenge in all nutrition research that requires participants to self-report their intake. Nevertheless, the validity of a multiple pass recall has been demonstrated in comparison to other subjective measures of dietary intake data (Moshfegh et al., 2008). A control group was not included, therefore, dietary change was compared pre- and post-intervention, within and between participants with a risk and non-risk associated genotype. The inclusion of a control group would have enabled us to discern the effect of gene-based dietary advice compared to general dietary advice. However, this comparison was not the aim of the current study. Participant numbers were low particularly in the *ApoE* risk group and those that were not meeting folate recommendations at baseline. Low participant numbers increase the risk of a type II error and may explain why a significant difference was not found in dietary change between risk and non-risk *MTHFR* participants who were not meeting folate recommendations. As in the Food4Me study (Fallaize et al., 2016), the rs429358 SNP in the *ApoE* gene was not in Hardy Weinberg equilibrium. However, haplotype frequencies in the present study (ɛ2, 6%; ɛ3, 82%; ɛ4, 12%) and participant profiles were similar to previous studies (Fallaize et al., 2016; Schiele et al., 2000). Health behaviour change is tasked with both initiation and maintenance of change, acquiring the motivation to change behaviour is an important step in the initiation of behaviour change (Ryan et al., 2008). The present study assessed the use of gene-based personalised nutrition advice to motivate the initiation of short-term dietary changes however, it is not possible to determine if these changes were maintained. Considering the attrition rate we observed after 10 days it is likely that the study would have been underpowered if the follow-up was extended. Previous studies have demonstrated significant dietary behaviour change 12 months after gene-based personalised recommendations (Horne et al., 2020; Nielsen and El-Sohemy, 2014) and in the longest follow-up to date that these changes can be observed more than 5 years after the intervention (Hietaranta-Luoma et al., 2018). The aim of our study was to use genotyping to promote adherence to associated general dietary recommendations. Participants were advised of their current intake and how it compared to the general UK recommendation for saturated fat and folate and their genotype and how that may interact with their diet to affect their risk of CVD. Previous studies have used personalised nutrition to provide individualised recommendations based on genotypes that have for example resulted in enhanced weight loss (Arkadianos et al., 2007; Vranceanu et al., 2020). This type of advice is currently being provided by numerous commercial companies (De et al., 2019). Providing more accurate individualised advice which over time provides individuals with greater success because of changes in dietary behaviour may result in greater maintenance of those behaviours. This would be an interesting area for future research in personalised nutrition to promote behaviour change.

### Conclusion

In conclusion, genotype-based personalised nutrition advice led to favourable dietary changes in participants who were not meeting dietary recommendations, irrespective of risk or non-risk genotype. In participants not meeting dietary recommendations, only those with a risk *ApoE* genotype met saturated fat recommendations following personalised nutrition advice. Therefore, incorporation of genotype-based personalised nutrition advice in a diet behaviour intervention may initiate favourable changes in dietary behaviour. Maintenance of positive dietary behaviours is essential to observe health benefits. Further research is required to determine the long-term effect of genotype-based personalised dietary advice on dietary behaviour and associated markers of health.

## Supplemental Material

sj-docx-1-nah-10.1177_02601060211032882 - Supplemental material for Does personalised nutrition advice based on apolipoprotein E and methylenetetrahydrofolate reductase genotype affect dietary behaviour? Click here for additional data file. Supplemental material, sj-docx-1-nah-10.1177_02601060211032882 for Does personalised nutrition advice based on apolipoprotein E and methylenetetrahydrofolate reductase genotype affect dietary behaviour? by Alexandra King, Shaghayegh Saifi, Jenna Smith, Leta Pilic, Catherine A-M Graham, Viviane Da Silva Anastacio, Mark Glaister and Yiannis Mavrommatis in Nutrition and Health
